# A machine learning-based algorithm used to estimate the physiological elongation of ocular axial length in myopic children

**DOI:** 10.1186/s40662-020-00214-2

**Published:** 2020-10-22

**Authors:** Tao Tang, Zekuan Yu, Qiong Xu, Zisu Peng, Yuzhuo Fan, Kai Wang, Qiushi Ren, Jia Qu, Mingwei Zhao

**Affiliations:** 1 Department of Ophthalmology & Clinical Centre of Optometry, Peking University People’s Hospital, Beijing, 100044 China; 2 College of Optometry, Peking University Health Science Center, Beijing, China; 3 Eye Disease and Optometry Institute, Peking University People’s Hospital, Beijing, China; 4 Beijing Key Laboratory of the Diagnosis and Therapy of Retinal and Choroid Diseases, Beijing, China; 5 Academy for Engineering & Technology, Fudan University, Shanghai, China; 6 Department of Biomedical Engineering, College of Engineering, Peking University, Beijing, 100871 China; 7 School of Ophthalmology and Optometry and Eye Hospital, Wenzhou Medical University, Wenzhou, Zhejiang China

**Keywords:** Myopia, Myopia progression, Machine learning, Ocular axial length, Physiological elongation, Orthokeratology

## Abstract

**Background:**

Axial myopia is the most common type of myopia. However, due to the high incidence of myopia in Chinese children, few studies estimating the physiological elongation of the ocular axial length (AL), which does not cause myopia progression and differs from the non-physiological elongation of AL, have been conducted. The purpose of our study was to construct a machine learning (ML)-based model for estimating the physiological elongation of AL in a sample of Chinese school-aged myopic children.

**Methods:**

In total, 1011 myopic children aged 6 to 18 years participated in this study. Cross-sectional datasets were used to optimize the ML algorithms. The input variables included age, sex, central corneal thickness (CCT), spherical equivalent refractive error (SER), mean K reading (K-mean), and white-to-white corneal diameter (WTW). The output variable was AL. A 5-fold cross-validation scheme was used to randomly divide all data into 5 groups, including 4 groups used as training data and one group used as validation data. Six types of ML algorithms were implemented in our models. The best-performing algorithm was applied to predict AL, and estimates of the physiological elongation of AL were obtained as the partial derivatives of *AL*_*predicted*_-age curves based on an unchanged SER value with increasing age.

**Results:**

Among the six algorithms, the robust linear regression model was the best model for predicting AL, with a *R*^*2*^ value of 0.87 and relatively minimal averaged errors between the predicted AL and true AL. Based on the partial derivatives of the *AL*_*predicted*_-age curves, the estimated physiological AL elongation varied from 0.010 to 0.116 mm/year in male subjects and 0.003 to 0.110 mm/year in female subjects and was influenced by age, SER and K-mean. According to the model, the physiological elongation of AL linearly decreased with increasing age and was negatively correlated with the SER and the K-mean.

**Conclusions:**

The physiological elongation of the AL is rarely recorded in clinical data in China. In cases of unavailable clinical data, an ML algorithm could provide practitioners a reasonable model that can be used to estimate the physiological elongation of AL, which is especially useful when monitoring myopia progression in orthokeratology lens wearers.

## Background

Myopia is currently the most common type of refractive error and has become a global problem as reported by population-based prevalence studies worldwide [1, 2]. According to epidemiological research, an unprecedented increase in myopia has been reported, especially in East Asia [3]. However, in recent years, the myopic population has become increasingly younger in China because of heavy near work and academic pressure [4]. If myopia develops at an early age and is not controlled in a timely manner, it is likely to develop into high myopia, which can cause a series of comorbidities, such as cataract, glaucoma and retinal complications, and increases the risk of severe and irreversible vision loss [5–7].

In recent years, increasingly more parents have sought myopia treatments with myopia control effects for their children, such as multifocal soft contact lenses [8], orthokeratology lenses (ortho-K lenses) [9, 10], specially designed spectacle lenses [11], and low-dose atropine eye drops [12]. In China, ortho-k lenses are more popular for treating myopic children because their effectiveness against myopia progression has been proven to be as high as 32–55% [9, 10], and they are more readily available in optometry clinics. During follow-ups, cycloplegic refraction and axial length (AL) measurements are useful tools used to evaluate the severity of myopia progression. However, for practitioners to judge the true extent of myopia progression in an ortho-K lens wearer, ortho-k treatment must be discontinued for at least 3–4 weeks before performing a cycloplegic refraction examination [13]. Alternatively, simply assessing the change in AL can be performed to evaluate myopia progression.

Clinically, axial myopia is the most common type of myopia in children [14], and myopia progression can be approximately estimated by AL elongation. Some researchers have studied how AL elongation influences myopia progression. On average, in young children, one diopter (D) of myopia is accompanied by an AL increase of approximately 0.3 to 0.5 mm [15, 16]. However, this elongation in AL does not always indicate myopia progression and could instead simply reflect physiological AL elongation (denoted as *∆AL*_*Phy*_), which is mainly compensated by a decrease in lens power in childhood [17]. Additionally, due to the significant difference in anterior chamber depth (ACD) among myopes, emmetropes, and hyperopes [18, 19], the deepening of the ACD may play a potential role in compensating *∆AL*_*Phy*_. The estimation of *∆AL*_*Phy*_ is especially useful for practitioners in evaluating myopia progression in myopic children who underwent ortho-k treatment as refraction examinations fail in an ortho-k lens wearer once the corneal curvature (CR) has changed. Previous studies provided information regarding deducing *∆AL*_*Phy*_. Tideman et al. [20] studied AL-age curves in European children aged 6 to 9 years and found that myopic children had an AL growth rate of 0.34 mm/year, which is more rapid than that of emmetropes (0.19 mm/year) and hyperopes (0.15 mm/year). Although the authors did not mention the concept of *∆AL*_*Phy*_, the 5th percentile of the AL-age curves representing those who did not develop myopia from age 6 to adulthood indicated an AL increase of only 0.8–0.9 mm [20]. Another study [21] conducted in Asian children suggested that for those with persistent emmetropes aged 7 to 12, the AL elongation over 5 years could be estimated as approximately 0.6 mm. However, the estimation of *∆AL*_*Phy*_ in Chinese children is particularly difficult because researchers cannot easily find groups of persistent emmetropes or myopic children who show no myopia progression during their growth.

Machine learning (ML) approaches, such as random forest, support vector machine (SVM), k-nearest neighbour, and decision trees, have been used to determine the prognosis of myopia and the diagnosis of glaucoma and age-related macular degeneration [22–25]. Compared with traditional approaches using regression-based algorithms, ML largely reflects a correlation analysis rather than predictive analytics [26] as a correlation analysis can be used to not only analyse and summarize complex datasets for the discovery of new knowledge but also improve predictive accuracy by exploiting complex interactions between predictors. Additionally, ML offers a good strategy to standardize predictive models that may address current limitations, including the linear and homogeneous nature of predictors [27]. In addition to its potentially improved predictive accuracy, ML can analyse latent variables, which are unlikely to be observed but may be inferred from other variables [28]. Thus, in this study, we aimed to construct an ML-based model for the estimation of *∆AL*_*Phy*_ in Chinese myopic children.

## Methods

### Subjects

**Fig. 1:**
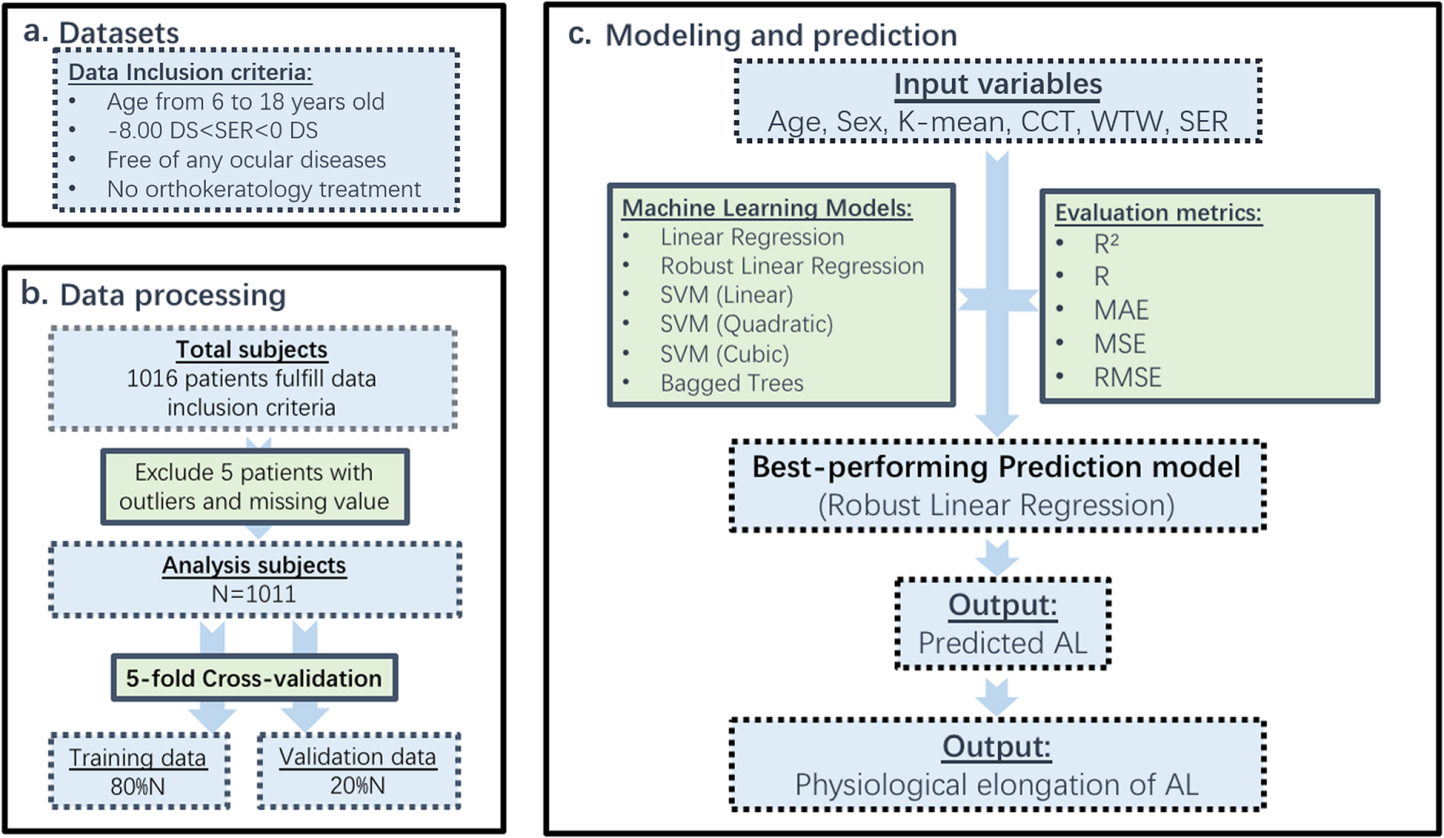
Flow chart of our proposed method. **a** Data inclusion criteria. **b** Data processing procedure. **c** Machine learning models used to predict the axial length and estimate the physiological axial length elongation. The best-performing prediction model was applied to predict the axial length and estimate the physiological axial length elongation by considering the partial derivatives of *AL*_*predicted*_-age curves. K-mean: mean K reading; CCT: central corneal thickness; ACD: anterior chamber depth; WTW: white-to-white corneal diameter; SER: spherical equivalent refraction error; AL: axial length; SVM: support vector machine; R: the coefficient of determination; MAEs: mean absolute errors; MSEs: mean squared errors; RMSE: root mean square error; N: number of patients

This retrospective study consisted of participants who visited our optometry centre due to myopia from January 2017 to December 2018. In total, 1016 participants underwent a comprehensive ophthalmological examination and fulfilled the inclusion criteria, which required a suitable age from 6 to 18 years, spherical equivalent refractive error (SER) ranging from 0 to − 8.00 D, astigmatism no greater than 3.00 D, the absence of any ocular diseases, and no history of orthokeratology treatment (Fig. 1). After excluding 5 patients because of extreme outlying observations (SER < − 8.00 D) or missing values, 1011 patients’ data were analysed. The purposes and procedures of this study were explained to the parents or legal guardians in detail, and they signed written informed consent forms for data storage and data usage for clinical/research purposes before the study, which was approved by the institutional research ethics committee of Peking University People’s Hospital and adhered to the tenets of the Declaration of Helsinki.

### Data collection and pre-processing

During the data collection process, only right-eye parameters were collected as individual sample data because of the high correlation between both eyes. The AL data were obtained with noncontact partial-coherence laser interferometry (IOL Master; Carl Zeiss Meditec, Oberkochen, Germany), and the other ocular biometry parameters were measured by a corneal topography system (SIRIUS SYSTEM, Italy). Previous studies have demonstrated that AL elongation is statistically and significantly associated with age, sex, the SER (SER = spherical degree + 0.5 × cylinder degree), mean K reading (K-mean; K-mean = (flat K reading + steep K reading)/2), central corneal thickness (CCT), and white-to-white corneal diameter (WTW) [14, 29–32]. To measure AL, at least five separate measurements were obtained per eye and were then averaged to obtain the mean AL value. For the other parameters, including the K-mean, ACD, CCT and WTW, three separate measurements were performed, and the average values were recorded. The spherical, cylindrical and SER data were based on the cycloplegic results, which were obtained 25 min after the instillation of three drops of 0.5% compound tropicamide eye drops (Santen Pharmaceutical Co. Ltd., Japan, 0.5% tropicamide combined with 0.5% phenylephrine) separated by a 5-min interval. Autorefraction was performed to measure the myopic refractive error in each subject. The ocular parameters are presented as the range (min to max) and the mean values ± standard deviation (mean ± SD).

### Pipeline of AL prediction and estimation of *∆AL*_*Phy*_

The pipeline of our research is shown in Fig. 1. In this study, to build the prediction model, the input variables were age (in years), sex (“1” represents male, while “0” represents female), K-mean (in diopters), ACD (in mm), CCT (in μm), WTW (in mm) and SER (in diopters), and the target output was the predicted AL (*AL*_*predicted*_, in mm). Six types of models were implemented in our study based on two types of linear regression models, three types of SVM regression models [33] and Bagged Trees model [34]. Then, a 5-fold cross-validation [35] scheme randomly divided all data into 5 groups, including 4 groups (80%) used as training data and one group (20%) used as validation data. This process was repeated 5 times such that all data were validated by this model, which allowed better prediction of the overall sample and prevented overfitting, and this type of pipeline was used to evaluate the performance of different models. During the fitting process, ACD was finally excluded from our final model because this parameter may substantially change with an increasing age [36]. Introducing ACD into the model could lead to the problem of collinearity and render the final model unsolvable. Furthermore, including ACD did not yield better results. Thus, the final model used to estimate AL was as follows:

1 $$ {AL}_{predicted}=f\left( Age,{K}_{mean}, CCT, WTW, SER, Sex\right) $$

To test the accuracy of this model, we classified the patients based on different SER, K-mean and sex, and the estimated AL error (*AL*_*error*_) was defined as follows:

2 $$ {AL}_{error}={AL}_{predicted}-{AL}_{true} $$

where *AL*_*true*_ is the true value of AL. To generate a specific *AL*_*predicted*_-age curve with unchanged SER, the K-mean, CCT, WTW, SER and Sex values should be fixed to constant values, and in this study, we set CCT as 550 μm [37, 38] and WTW as 12 mm for illustration [39, 40]. The SER values were set to different constant values as − 1.00 D, − 2.00 D, − 3.00 D, − 4.00 D, − 5.00 D and − 6.00 D. The K-mean values were set as 40.00 D, 42.00 D, 44.00 D and 46.00 D. Each *AL*_*predicted*_-age curve represents how AL increases with age, while SER does not change.

Theoretically, the physiological AL elongation from Age1 to Age2 (*∆AL*_*phy*(*Age*2,  *Age*1)_) i.e., from 6 to 8 years, can be calculated as follows:

3 $$ {\Delta  AL}_{phy\left( Age2,\kern0.5em Age1\right)}=f(Age2)-f(Age1) $$

To obtain a general solution, we can define the rate of predicted AL elongation (*∂AL*_*Phy*_) by considering the partial derivatives of the AL-age curves as follows:

4 $$ {\partial AL}_{Phy}={f}_{Age}^{\prime}\left( Age,{K}_{mean}, CCT, WTW, SER, Sex\right)=\frac{\partial AL}{\partial Age} $$

Additionally, *∆AL*_*phy*(*Age*2,  *Age*1)_ could be further written as follows:

5 $$ {\Delta  AL}_{phy\left( Age2,\kern0.5em Age1\right)}=f(Age2)-f(Age1)={\int}_{Age1}^{Age2}{f}_{Age}^{\prime }(Age) dAge $$

To explore the mechanism underlying physiological AL elongation, the lens power was calculated using the Bennett-Rabbetts method [41–43].

### Statistical analysis

To verify the efficacy of the machine learning models, a multiple linear regression model was also created, and some traditional statistical methods were used for comparison. Paired *t*-tests were performed to compare *AL*_*predicted*_ and *AL*_*true*_ where the datasets were categorized into different subgroups based on SER (0 to − 3.00 D, − 3.00 D to − 6.00 D, and < − 6.00 D), K-mean (< 42.00 D, 42.00 D to 44.00 D, and > 44.00 D), age (6–10, 11–14, and 15–18 years) and sex (male or female), and the 95% confidence intervals (CIs) were estimated for the error. Pearson correlation analyses among the lens power, ACD, K-mean and age were performed. The performance of the machine-learning prediction algorithms developed from the training cohort was assessed using the validation cohort by calculating the *R*^*2*^ value, *R* value, mean absolute error (*MAE*), mean squared error (*MSE*), and root mean square error (*RMSE*), and all these linear regression indices were calculated using the MATLAB 2018a software package (The MathWorks, Inc., US), which was also used for all the ML experiments. The data obtained were analysed by the SPSS statistical software package (Version 22.0, IBM Corp., US), and the level of statistical significance was set at *P* < 0.05.

## Results and analysis

### Model analysis

In total, 491 males (48.57%) and 520 females (51.43%) were included in this study. The average age of all subjects was 11.18 ± 2.49 years. The mean SER was − 3.21 ± 1.61 D and ranged from 0 to − 8.00 D. The average AL was 24.95 ± 0.99 mm and ranged from 21.77 to 29.84 mm. The detailed information is provided in Table 1.

**Table 1:** Basic information and ocular parameters of the myopic subjects included in this study

Subjects	Values	
No. of cases	1011	
Sex, male No. (%)	491 (48.57)	
Sex, female No. (%)	520 (51.43)	
Parameters	Range	Mean ± SD
Age (years)	6–18	11.18 ± 2.49
ACD (mm)	2.51–4.23	3.33 ± 0.22
CCT (um)	448–688	553 ± 0.03
SER (D)	-8.00 - 0	−3.21 ± 1.61
K-mean (D)	38.26–47.99	43.33 ± 1.44
WTW (mm)	10.28–14.17	11.98 ± 0.44
AL (mm)	21.77–29.84	24.95 ± 0.99

*ACD* = anterior chamber depth; *CCT* = central corneal thickness; *SER* = spherical equivalent refraction error; *K-mean* = mean K reading; *WTW* = white-to-white corneal diameter; *AL* = axial length; *D* = diopters; *SD* = standard deviation

Significant correlations were found between the input variables and AL, and the *R*^2^ value of the multiple linear regression model was 0.81 as determined by the following equation:

6 $$ {AL}_{predicted}\kern0.5em =\kern0.5em 40.31+\left(0.056\times \mathrm{Age}\right)-\left(0.013\times \mathrm{Sex}\right)-\left(0.396\times {\mathrm{K}}_{\mathrm{mean}}\right)-\left(0.353\times \mathrm{SER}\right)\ \left(P<0.05\right) $$

Table 2 shows the performance results of six ML algorithms and the multiple linear regression model. The results show that most ML models had a predictive ability that surpassed that of the traditional statistical regression model, and the two linear ML models [44] and one SVM model achieved better performance. The relationships between AL and the other variables are linearly dependent since the ML algorithm used a linear regression, and the SVM with a linear kernel function also achieved relatively good results. More complicated models, such as an SVM with quadratic and cubic kernel functions, cannot achieve good performance since such higher-order models could generate overfitting and cannot be applied for our application.

**Table 2:** Performance of the machine learning algorithms and multiple linear regression model

	Algorithms	*R*^2^	*R*	RMSE	MAE	MSE
Traditional Statistical Method	Multiple Linear Regression	0.81	0.8985	0.4380	0.3455	0.1919
Machine Learning Methods	Linear Regression (linear)	0.86^*^	0.9276^*^	0.3782	0.2933	0.1430
	Linear Regression (Robust)	0.86^*^	0.9276^*^	0.3780^*^	0.2929	0.1427^*^
	SVM (linear)	0.86^*^	0.9276^*^	0.3781	0.2928^*^	0.1429
	SVM (Quadratic)	0.85	0.9219	0.3916	0.3013	0.1533
	SVM (Cubic)	0.82	0.9055	0.4291	0.3263	0.1841
	Bagged Trees	0.77	0.8775	0.4820	0.3583	0.2323

*SVM* = support vector machine; *RMSE* = root mean square error; *MAE* = mean absolute error; *MSE* = mean squared error

Best values of indices are marked by an asterisk (*)

**Fig. 2:**
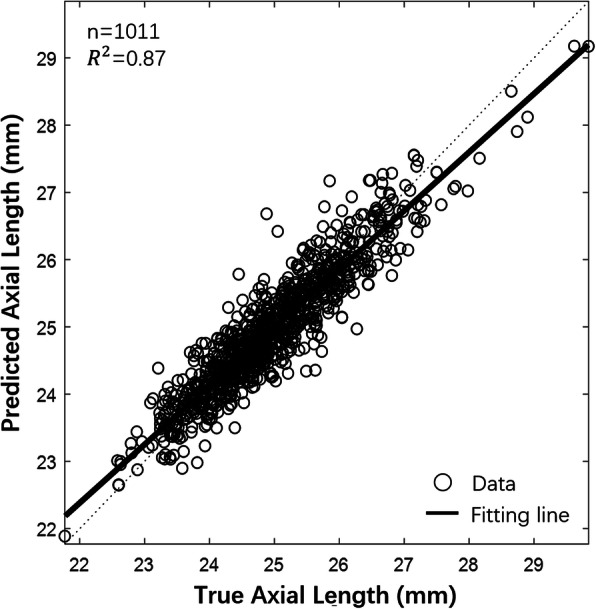
Final axial length prediction using machine learning with baseline input variables. Scatterplot of the predicted axial length vs. the true axial length. The solid line represents the perfect correlation regression line. The dashed line represents the perfect line without error prediction

Our results reveal that the ML model that used the robust linear regression algorithm effectively predicted AL, and the four performance indices achieved the best values in this study (Table 2). As shown in Fig. 2, most scatterplots fall along the perfect correlation regression line (*r* = 0.912, *P* < 0.0001, *R*^2^ = 0.87), indicating an excellent correlation between the predicted values and the true values. In addition, the numbers of overestimated and underestimated values are limited.

### Prediction accuracy

In our models, when the input variables are changed, the *AL*_*predicted*_ changes accordingly. As shown in Table 3, the error and 95% CI fluctuated within very narrow ranges. There were no significant differences between the means of *AL*_*predicted*_ (24.95 ± 0.99 mm) and *AL*_*true*_ (24.95 ± 0.91 mm) of the whole sample (t = 0.007, *P* = 0.994), and paired *t*-tests showed no significant differences among the different subgroups (all *P* > 0.05), indicating that the robust linear model achieved a high level of precision.

**Table 3:** The means of the predicted axial length vs. the true axial length

Groups	SER (D)			K-mean (D)			Age (years)					
	0 to − 3.00	−3.00 to − 6.00	< − 6.00	< 42.00	42.00 to 44.00	> 44.00	6–10		11–14		15–18	
							M	F	M	F	M	F
No. of cases (%)	518 (51.2)	413 (40.9)	80 (7.9)	177 (17.5)	515 (50.9)	319 (31.6)	219 (21.7)	241 (23.8)	206 (20.4)	221 (21.8)	66 (6.5)	58 (5.8)
Predicted AL (mm)	24.46	25.25	26.47	25.78	25.01	24.38	24.57	24.56	25.12	25.12	25.69	25.74
True AL (mm)	24.46	25.27	26.44	25.82	25.01	24.37	24.56	24.59	25.12	25.16	25.71	25.64
Error (mm)	0	−0.02	0.03	− 0.04	0	0.01	0.01	−0.03	0	−0.04	− 0.02	0.10
95% CI for error (mm)	[−0.03, 0.03]	[−0.02, 0.06]	[− 0.07, 0.14]	[− 0.10, 0.03]	[−0.03, 0.03]	[− 0.03, 0.04]	[− 0.04, 0.05]	[−0.07, 0.02]	[− 0.05, 0.06]	[− 0.08, 0.01]	[−0.11, 0.07]	[− 0.01, 0.22]
95% CI for AL (mm)	[23.72, 25.18]	[24.81, 26.73]	[24.50, 30.14]	[23.16, 26.47]	[24.19, 25.82]	[23.54, 25.41]	[23.60, 25.92]	[22.82, 25.20]	[23.85, 26.59]	[23.10, 25.48]	[22.83, 27.52]	[25.39, 31.34]
*P* value	0.457	0.234	0.420	0.372	0.933	0.371	0.774	0.991	0.951	0.328	0.962	0.256

*SER* = spherical equivalent refraction error; *K-mean* = mean K reading; *AL* = axial length; *D* = diopters; *CI* = confidence interval; *M* = male; *F* = female

Based on spherical equivalent refraction error (SER), mean K reading (K-mean), age and sex distribution of all samples

### Prediction results

**Fig. 3:**
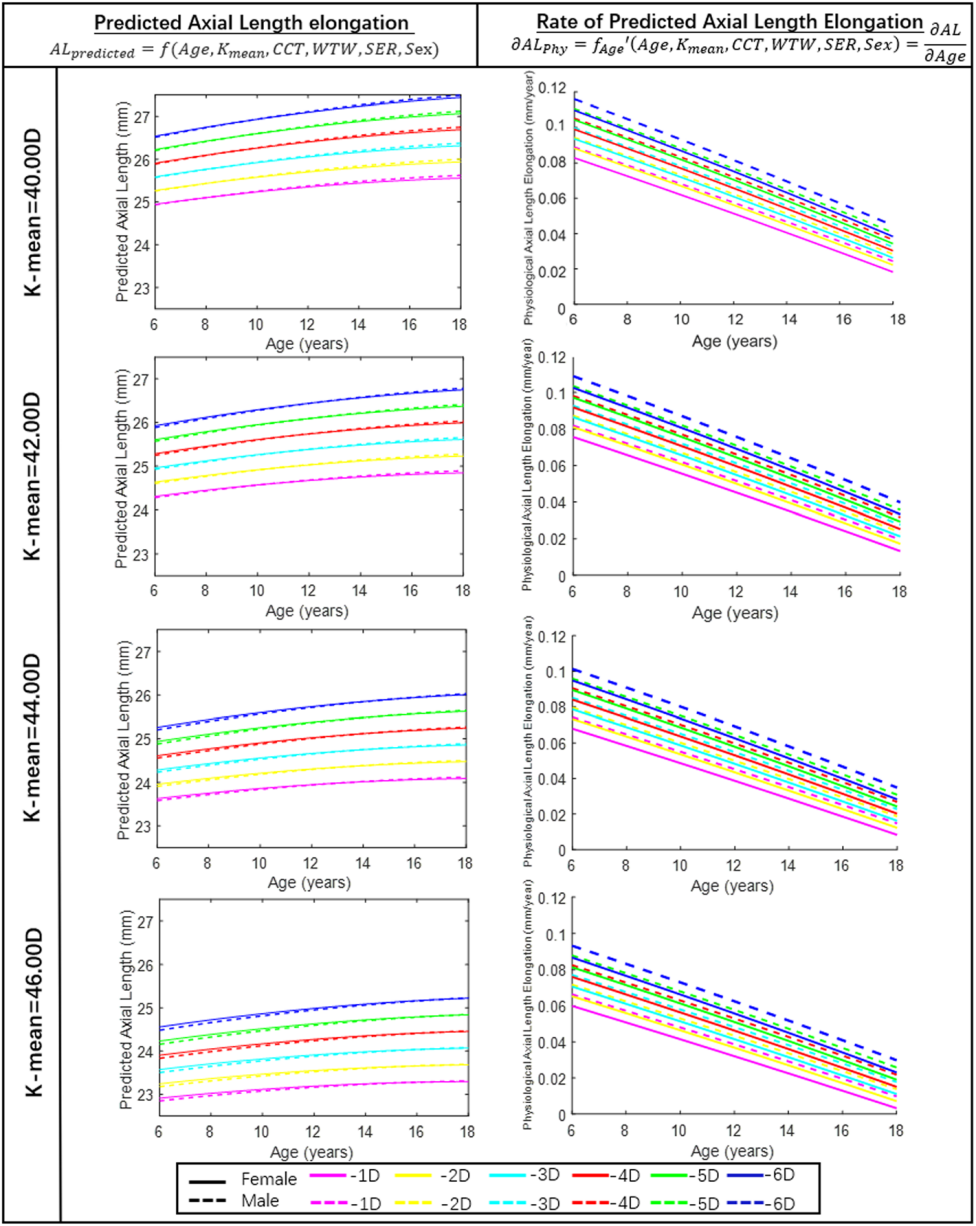
Growth curves of predicted axial length elongation vs. age and rate of predicted axial length elongation vs. age. Left panel: Growth charts (predicted axial length elongation vs. age). Right panel: Growth charts (rate of predicted axial length elongation vs. age) with the spherical equivalent refraction error fixed at − 1.00 D, − 2.00 D, − 3.00 D, − 4.00 D, − 5.00 D and − 6.00 D and the mean K reading fixed at 40.00 D, 42.00 D, 44.00 D and 46.00 D. Males are indicated by dashed lines, and females are indicated by solid lines

The left panel of Fig. 3 shows the *AL*_*predicted*_-age curves based on different SER values (the SER value was fixed as − 1.00 D, − 2.00 D, − 3.00 D, − 4.00 D, − 5.00 D and − 6.00 D) and K-means (fixed as 40.00 D, 42.00 D, 44.00 D and 46.00 D) for both sexes. From ages 6 to 18, all *AL*_*predicted*_ presented an increasing trend with unchanged SER values, supporting the notion that the elongation of AL does not always result in myopia progression. The subjects with a smaller K-mean value demonstrated greater *AL*_*predicted*_ as their age increased, while those with a greater K-mean experienced smaller *AL*_*predicted*_. Based on the estimation, AL elongation was greater in males than females in all age groups.

The right panel of Fig. 3 shows the estimations of *∂AL*_*Phy*_ with increasing age considering sex. A more myopic SER corresponded to greater *∂AL*_*Phy*_. However, a larger K-mean corresponded to smaller *∂AL*_*Phy*_, and the results showed that the mean *∂AL*_*Phy*_ in males was slightly larger than that in females. For example, in 6-year-old myopic children, the mean *∂AL*_*Phy*_ was predicted to be approximately 0.092 mm/year (from 0.066 mm/year to 0.116 mm/year) in males and approximately 0.085 mm/year (from 0.060 mm/year to 0.110 mm/year) in females, and these values decreased linearly to approximately 0.027 mm/year (from 0.010 mm/year to 0.045 mm/year) in males and approximately 0.021 mm/year (from 0.003 mm/year to 0.038 mm/year) in females among the 18-year-old young adults with myopia. More detailed estimations of *∂AL*_*Phy*_ are shown in Table 4. Although the estimated values of *∂AL*_*Phy*_ are relatively small, an integral in Eq. (5) larger than 1 mm could be expected over a longer period.

**Table 4:** Estimations of physiological elongation of axial length (in mm/year) for 6-year-old and 18-year-old males and females

	SER (D)													
K-mean	-1.00		-2.00		-3.00		-4.00		-5.00		-6.00		Mean	
(D)	Age (years)													
	6	18	6	18	6	18	6	18	6	18	6	18	6	18
40.00 (M)	0.089	0.024	0.094	0.028	0.100	0.032	0.105	0.037	0.111	0.041	0.116*	0.045	0.103	0.034
40.00 (F)	0.083	0.018	0.088	0.022	0.094	0.026	0.099	0.030	0.104	0.034	0.110*	0.038	0.096	0.028
42.00 (M)	0.082	0.020	0.087	0.024	0.098	0.032	0.098	0.032	0.104	0.036	0.109	0.040	0.095	0.030
42.00 (F)	0.076	0.013	0.081	0.017	0.086	0.021	0.092	0.025	0.097	0.029	0.103	0.033	0.089	0.023
44.00 (M)	0.074	0.015	0.080	0.019	0.085	0.027	0.091	0.027	0.096	0.031	0.101	0.035	0.089	0.025
44.00 (F)	0.068	0.008	0.073	0.012	0.079	0.016	0.084	0.020	0.090	0.024	0.095	0.028	0.081	0.018
46.00 (M)	0.066	0.010*	0.072	0.014	0.077	0.022	0.083	0.022	0.088	0.026	0.093	0.030	0.080	0.020
46.00 (F)	0.060	0.003*	0.065	0.007	0.071	0.011	0.076	0.015	0.081	0.019	0.087	0.023	0.073	0.013
Mean (M)	0.078	0.017	0.083	0.021	0.089	0.029	0.094	0.029	0.100	0.033	0.105	0.037	0.092	0.027
Mean (F)	0.072	0.011	0.077	0.015	0.082	0.019	0.088	0.023	0.093	0.027	0.099	0.031	0.085	0.021

The maximum and minimum values for females and males are marked by an asterisk (*). The spherical equivalent refraction error (SER) were fixed at -1.00D, -2.00D, -3.00D, -4.00D, -5.00D and -6.00D and the mean K reading (K-mean) were fixed at 40.00D, 42.00D, 44.00D and 46.00D

*SER* spherical equivalent refraction error, *K-mean* mean K reading, *M* male, *F* female, *D* diopter

### Correlation between the input variables and subject age

The lens powers were estimated in all subjects based on both the Modified Stenstrom [45] and Bennett-Rabbetts methods [41–43] using Gullstrand-Emsley and Bennett-Rabbetts eye models [46], and a third customized eye model was applied based on customized c constants (Table 5). Table 6 shows the lens powers calculated using the Bennett-Rabbetts method with the customized c constants and the changes in AL in different age groups. The differences in the lens powers among all age groups were statistically significant (*P* < 0.01).

**Table 5:** Calculated lens powers using the biometry and phakometry data of the whole population

Method	Symbol	Eye Model	***c constants*** (mm)	Average (D)
Modified Stenstrom	P_L,Sten_	Gullstrand-Emsley	c_Sten_ = 2.145	20.68 ± 1.44
		Bennett-Rabbetts	c_Sten_ = 2.221	20.82 ± 1.45
		Customized	c_Sten_ = 2.875 ± 0.763	22.07 ± 1.56
Bennett-Rabbetts	P_L,BR_	Gullstrand-Emsley	c_BR_ = 2.230	22.34 ± 1.54
		Bennett-Rabbetts	c_BR_ = 2.306	22.52 ± 1.56
		Customized	c_BR_ = 2.891 ± 0.778	23.95 ± 1.68

*n* = 1011 eyes; *D* = diopters

**Table 6:** Lens power calculations in different age groups using the Bennett-Rabbetts method (customized)

Age groups (years)	No. of cases (%)	P_L,BR_ Customized (D)	AL (mm)	*P* value
6–9	293 (28.98)	24.66 ± 1.57	24.53 ± 0.91	< 0.01
10–12	431 (42.63)	23.91 ± 1.63	24.87 ± 0.85	< 0.01
13–15	224 (22.16)	23.40 ± 1.53	25.36 ± 0.92	< 0.01
16–18	63 (6.23)	22.90 ± 1.63	25.92 ± 1.30	< 0.01

*AL* = axial length; *D* = diopters

**Fig. 4:**
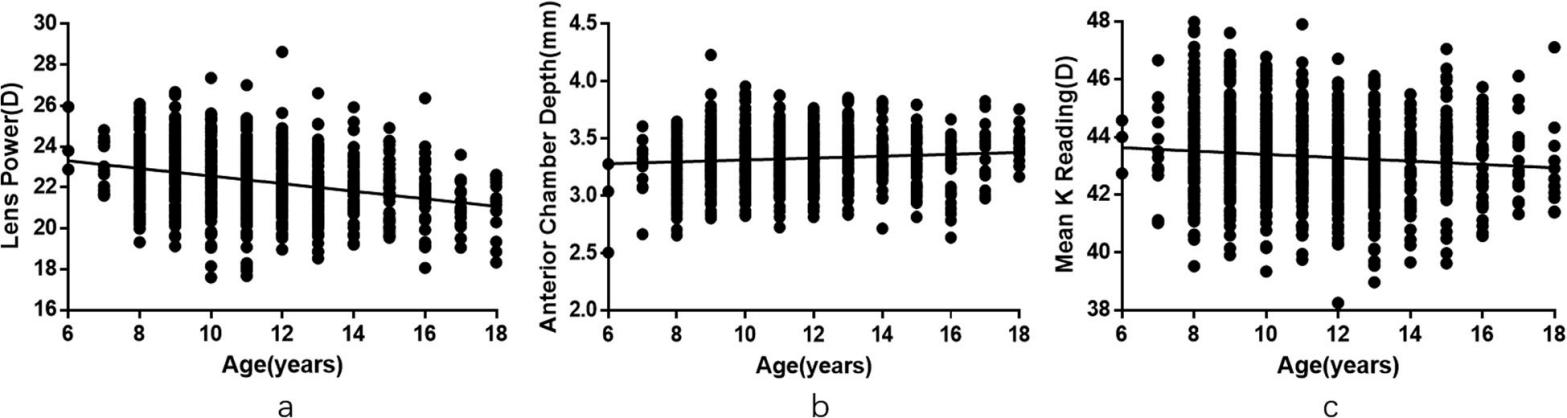
Scatterplots of the calculated lens powers, anterior chamber depth, mean K reading and age. **a** Calculated lens powers vs. age. **b** Anterior chamber depths vs. age. **c** Mean K readings vs. age. The lens power and K-mean were negatively correlated with age, while ACD was positively correlated with age

The scatterplots shown in Fig. 4 illustrate that the lens power and K-mean were negatively correlated with age (lens power: *r* = − 0.301, *P* < 0.01; K-mean: *r* = − 0.125, *P* < 0.01), while ACD was positively correlated with age (*r* = 0.093, *P* < 0.01).

## Discussion

Increased AL does not always indicate myopia progression in children. Clinically, a small amount of AL elongation often results in no change in the SER in myopic children and could be compensated by changes in the lens power, ACD or even CR. However, CR, CCT and WTW are relatively stable parameters with increasing age in children [37–40]; thus, *∂AL*_*Phy*_ could be estimated by considering the partial derivatives of *AL*_*predicted*_-age curves based on unchanged SER values and these input variables, and *∆AL*_*phy*(*Age*2,  *Age*1)_ could be calculated by Eq. (5). In clinical observations, although the amount of AL elongation each year can be measured by an IOL Master or A-scan ultrasound device [20], this physiological AL elongation per year can hardly be recorded due to the severe situation of myopia progression in China in which almost all myopic children show myopia progression every year ranging from − 0.25 D/year to as high as − 2.00 D/year, which we called a “myopia boom”, with an incidence of more than 95.5% among university students and 84.6% among school children in large Chinese cities [47–49]. This situation complicates the identification of a group of children aged 6 to 18 years who are non-myopic and creates difficulty in maintaining complete records of these children’s physiological AL elongation. Another concern is that in myopic children undergoing ortho-K treatment, it is difficult for practitioners to determine the progression of the SER based only on elongated AL data unless the physiological component was defined; therefore, we applied ML-based algorithms to estimate *∆AL*_*phy*(*Age*2,  *Age*1)_.

The estimation of the physiological component of increases in AL was based on the precise prediction of AL according to biometric parameters, including age, sex, CCT, SER, K-mean, and WTW. Then, *∂AL*_*Phy*_ could be estimated from the partial derivatives of *AL*_*predicted*_-age curves based on an unchanged SER. Despite the small sample in this study, we found a relatively high prediction accuracy for AL using the robust linear regression model, which was characterized by its resistance to deviations from the common distributional assumptions and facilitated improvements in inferences in the presence of outliers [50]. Compared with conventional models or traditional statistical methods, the robust linear regression model has a faster modelling speed and does not require very large sample sizes and complicated calculations. In addition, the model can run rapidly even with a large amount of data and provide an understanding and interpretation of each variable according to the coefficient. Furthermore, the model has a higher sensitivity to outliers and, thus, enables more reliable inferences. Based on the simulation results above, the ML algorithm using the robust model provided reliable results and should be considered a favourable tool for predicting both AL and the physiological elongation of AL.

### *AL*_*predicted*_-age curves deduced by the ML algorithm

Some studies have demonstrated that AL plays a significant role in the determination of the refractive status and that the loss of refractive power from the cornea and the lens compensates for AL elongation, resulting in a relatively stable SER with increasing age [51]. Once the balance is disrupted, myopia develops if the rate of AL elongation outpaces the reduction in the corneal and lens power [52, 53]. Therefore, faster AL elongation corresponds to a greater possibility that a subject will become more myopic at an early age.

In an epidemic study performed by Diez et al. [54], the prevalence of myopia increased with age, while the prevalence of hyperopia decreased. However, some subjects below the first quartile showed a stabilized AL after the age of 12 years. As deduced from their study, the children over the age of 12 who did not show significant myopia progression tended to have considerably flatter AL growth curves, which coincides with our findings. Another recent study reported similar results [20] indicating that AL stabilization occurred as early as 14 years. As indicated in Fig. 3 (left charts), the *AL*_*predicted*_-age curve obtained by the ML algorithm gradually slowed and was essentially stable with increasing age in both male and female subjects.

In the clinic, an ortho-K practitioner can evaluate the severity of myopia progression without ceasing lens wear by calculating the difference between the true AL elongation (*∆AL*_*true*_) and *∆AL*_*phy*(*Age*2, *Age*1)_. If a child had a value of *∆AL*_*phy*(*Age*2,  *Age*1)_ equal to *∆AL*_*true*_, one can conclude there is no myopia progression. However, because ortho-K lens treatment could result in the thinning of the central corneal epithelium [55] and thickening of the choroid [56], which may result in a “shortening” of the AL, practitioners must perform all AL measurements after ortho-K treatment to calculate *∆AL*_*true*_ while using the parameters sampled before the ortho-K treatment to estimate *∆AL*_*phy*(*Age*2,  *Age*1)_. For example, consider the case of an eight-year-old myopic child who underwent ortho-K treatment. After 3 weeks of overnight lens wear, an AL measurement should be performed as baseline data, while another AL measurement should be performed 1 year later. The difference between these two measurements is *∆AL*_*true*_, while *∆AL*_*phy*(9,  8)_ could be estimated by the ML model for comparison.

### Factors influencing *∂AL*_*Phy*_

Some association exists between CR and AL. A previous study indicated that myopia showed a linear relationship with the flattening of the cornea with increasing AL [29]. However, another study showed that the CR was not strongly related to AL [57]. As shown by our findings, a flattened cornea was associated with a longer AL under the same degree of myopia. Based on the prediction model, we also calculated the relationship between *∂AL*_*Phy*_ and the CR. Although Zadnik et al. reported that the corneal power was minimally altered during the school years, the CR of emmetropic children was flattened by 0.06 D at the age of 14 compared with that at age 6 [58]. Many studies have indicated no significant change in CR during the school years, which was the basis of the assumption of our model. In addition, a study by Tideman et al. revealed that myopic children had a flatter CR than both emmetropic and hyperopic children [20]. Our investigation shows that a steeper cornea is associated with less *∂AL*_*Phy*_ (Fig. 3), suggesting that a small amount of AL elongation resulting in myopia progression in a subject with a steep cornea may not cause SER change in a subject with a flat cornea, who has much better tolerance for the elongation of AL.

Children with myopia have a longer AL than emmetropes [31]. Mutti et al. [59] studied 605 children aged 6 to 14 years of different ethnicities and found that the AL of emmetropes increased at a steady rate of approximately 0.10 mm/year, while the myopic children exhibited greater AL elongation rates (0.10 to 0.17 mm/year). Similar findings were found in another study involving Asian children conducted by Wong et al., who showed an average AL elongation rate of approximately 0.12 mm/year in persistent emmetropic children aged 7 to 12 years, while the values in the persistent hyperopic and myopic children were approximately 0.10 and 0.31 mm/year, respectively [21]. Generally, the eye growth rate in myopic children is greater than that of emmetropic children, while the rate in hyperopic children is lower than that in emmetropic children. For comparison, in studies by Tideman et al. [20] and Mutti et al., the eye growth rate per year in emmetropic children could be considered the physiological elongation of AL, which was slightly greater than the value of *∆AL*_*phy*(*age* + 1, *age*)_ estimated in our study. Similar to the fact that different eye growth rates correlate with different refractive statuses, *∂AL*_*Phy*_ and *∆AL*_*Phy*_ were also influenced by myopia severity. The ML model showed that both predicted AL elongation and *∂AL*_*Phy*_ were negatively correlated with the SER, suggesting that a higher degree of myopia corresponded to greater *∆AL*_*phy*_ and that compared with subjects with low myopia, in subjects with high myopia, the increase in AL seems to have a lower effect on SER. To date, few studies provided clear evidence supporting this speculation. One possible explanation is that longer AL is often associated with flatter corneas [60], which were proven to be associated with larger *∆AL*_*phy*_ in our study. Given the limited evidence, further studies are still needed to prove this inference.

Several studies in recent years have demonstrated that AL is associated with age based on multivariate analyses [31, 61]. Our model also proves that AL increases with age. Other studies have shown similar findings [30, 32, 62]. As reported by Tideman et al., AL in the 95th percentile increased by 2.5 mm from 6 years of age to adulthood [20], and Diez et al. found that from 6 to 15 years of age, AL in the 95th percentile increased by 2.93 mm in females and 2.81 mm in males [54]. These values of AL elongation were longer than the values estimated in this study because these values included both the physiological and non-physiological components. As defined by the calculations, *∂AL*_*Phy*_ constitutes the partial derivatives of *AL*_*predicted*_-age curves under the assumption of an unchanged SER, and age is the most important factor that may influence *∂AL*_*Phy*_, which decreases as a child develops into a young adult, indicating that a 6-year-old child experiences much greater physiological AL elongation than an 18-year-old individual.

The absolute values of AL differ between males and females, and our model agrees with several previous investigations. Twelker et al. reported that the AL of 6- to 12-year-old children was elongated with increasing age and that the AL of males was 0.5 mm longer than that of females [63]. Diez et al. also revealed that females had significantly shorter ALs than males on average [54]. However, few studies have reported differences in physiological AL elongation between the sexes. In our study, as illustrated in Fig. 3, males had greater physiological elongation of AL than females, suggesting that males can tolerate more AL elongation without myopia progression than females.

### Limitations

The true values of the physiological elongation of ocular AL per year were clinically unavailable because of severe myopia progression in children in China. The decreasing partial derivatives of the AL elongation curves correlated well with clinical experience. However, the estimations of the physiological AL elongation were based on the assumption that the ocular parameters, including WTW, CCT and K-mean, did not significantly change with increasing age, and the estimations were the mean values of populations rather than individuals. Although the fitting results were excellent, the sample for ML was not large (1011 subjects), and a large amount of the patients’ data were centralized in the middle range, yielding an uneven distribution with a negative effect on the prediction accuracy. Compared with the numbers of subjects in the other age groups, few subjects were aged 16 to 18 years (accounting for 12.27% of the total sample). Another concern is the refractive status of populations. To obtain better knowledge regarding AL growth curves and how AL growth affects myopia progression, data from hyperopic and emmetropic subjects should be included in future work. Finally, the effects of genetic and environmental factors, such as outdoor activity, near work, or genetic backgrounds, were not included in our final models because of difficulties in introducing these factors as quantitative variables. Despite these limitations, the ML algorithm can provide researchers with a powerful tool to reasonably estimate *∆AL*_*Phy*_ or *∂AL*_*Phy*_ in different age groups of myopic children, which is useful for evaluating myopia progression under situations in which myopic children cannot easily undergo a cycloplegic refraction test, especially for ortho-K lens practitioners. Additionally, a customized software package or application could be easier for clinical practitioners to calculate and compare *∆AL*_*Phy*_ with *∆AL*_*true*_. Alternatively, practitioners could use Eq. (6) for a simplified process. Regardless of which model is chosen, it is important to use the AL measurement obtained after 3 weeks of ortho-K lenses wear and the AL measurements obtained in follow up visits to calculate and compare *∆AL*_*true*_ with *∆AL*_*Phy*_ because of changes in CR and choroidal thickness.

## Conclusions

The results of the present study verify that the ML algorithm using a robust linear regression model was better in predicting AL and estimating physiological AL elongation in a sample of Chinese school-aged myopic children based on only routine cross-sectional clinical data. Our study demonstrates the possibility that the physiological component of AL elongation can be estimated by ML algorithms. Based on the model, we can easily separate the amount of the non-physiological component from AL elongation, and myopia progression in children who underwent ortho-K treatment could be assessed without discontinuing lens treatment.

## Data Availability

The datasets used and/or analysed during the current study are available from the corresponding author upon reasonable request.

## Abbreviations

ML: Machine learning
AL: Axial length
SER: Spherical equivalent refraction error
ACD: Anterior chamber depth
CCT: Central corneal thickness
K-mean or K_mean_: Mean K readings
WTW: White-to-white corneal diameter
CR: Corneal curvature
D: Diopters
SVM: Support vector machine
RMSE: Root mean square error
MAE: Mean absolute error
MSE: Mean square error
CI: Confidence interval
*AL*_*predicted*_: Predicted axial length
*AL*_*error*_: Estimated axial length error
*AL*_*true*_: True axial length
*∂AL*_*Phy*_: Rate of predicted axial length elongation
*∆AL*_*phy*_: Physiological axial length elongation
*∆AL*_*phy*(*Age*2, *Age*1)_: Physiological axial length elongation from Age1 to Age2
